# Coyotes Choose Cover Over Concrete When Selecting Den Sites

**DOI:** 10.1002/ece3.73186

**Published:** 2026-02-27

**Authors:** Summer Fink, Daniela Guerrero, Eden Nitza, Michel Kohl

**Affiliations:** ^1^ Warnell School of Forestry and Natural Resources University of Georgia Athens Georgia USA; ^2^ Florida Fish and Wildlife Conservation Commission Tallahassee Florida USA

**Keywords:** coyote, den site selection, dens, fitness, reproductive decision making

## Abstract

Animal decision‐making directly impacts survival and reproductive success, particularly for reproductive habitat specialists (e.g., denning species) in highly dynamic environments. Despite the widespread and increasing distribution of coyotes ( *Canis latrans* ) in urban areas, little research has focused on how they select urban den sites. In this study, we investigated den structure and site selection of coyotes in Atlanta, Georgia. From January to April of 2022–2024, we GPS collared 48 coyotes and located 20 dens in April of 2022–2025. We measured the physical characteristics of dens and categorized structure type as anthropogenic or natural. We used a 3rd‐order resource selection analysis to assess impacts of impervious surface (%), distance to buildings, and distance to cover habitat on den site selection. We found that approximately half of the dens were located in anthropogenic structures, which coyotes appeared to use opportunistically for concealment and protection. We also identified strong selection for cover habitat and avoidance of impervious surfaces by coyotes for den sites. Our findings indicate that coyotes in Atlanta avoid areas with high human infrastructure, select areas with cover, and show flexibility in structure use allowing them to safeguard dens. While this showcases the adaptability of coyotes when making complex reproductive decisions, it also highlights the potential population level impacts increased urbanization and land conversion may have on urban wildlife.

## Graphical abstract

Reproductive decision‐making impacts fitness, especially in dynamic urban environments. Thus, we assessed urban coyote den site selection in Atlanta, GA. We found that coyotes made decision on both the site and structure of dens to mitigate fitness related risk and safeguard their young.

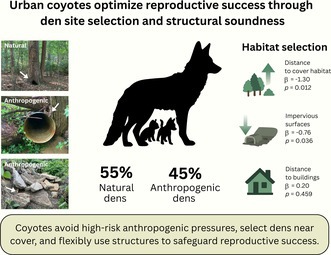

## 1 Introduction

To maximize survival and reproduction, wildlife must make decisions that weigh the costs and benefits of many interacting biological, environmental, and life history constraints (i.e., fitness optimization; Owen et al. 2017; Parker and Smith 1990; Reimer et al. 2019). To combat fitness related risks with reproductive decision‐making, some species have evolved restrictive reproductive life histories, such as site fidelity (Switzer 1993), philopatry (Williams and Rabenold 2005), and reproductive habitat specialization (Larson et al. 2020). Adaptive reproductive decisions, such as habitat specializations, play a key role in the survival of individuals at early life stages (Gibson et al. 2016). Thus, for wildlife species that are tightly tied to specific reproductive sites (e.g., dens, nests, calving grounds), decisions pertaining to site location can have implications beyond individual‐level fitness, specifically for species where early life history stages are critical for population trajectories (Tuljapurkar et al. 2009). For instance, historic wolf (*Canis* spp.) management used these sites as a tool for reducing both wolf abundance and densities (Jęodrzejewska et al. 1996). Similarly, caribou ( *Rangifer tarandus* ) prioritize lower predation risk as opposed to food availability when selecting calving grounds (Pinard et al. 2012) as calf survival is a main driver of their population dynamics (Boulanger et al. 2011). However, with today's rapidly changing environments, obligate reproductive traits may become maladaptive (Caspi et al. 2022), emphasizing the importance of reproductive decision‐making on both individual level fitness and population dynamics.

Anthropogenic pressure, such as pollution, habitat fragmentation, and land use change, can exacerbate challenges wildlife face in reproductive decision‐making (Owen et al. 2017). This is perhaps most pronounced in urban areas as they are some of the most dynamic decision‐making landscapes on the planet (Lee and Thornton 2021). The built environment, comprised of various forms of impervious surfaces, poses significant levels of risk that wildlife must navigate. For instance, paved surfaces, such as roads and parking lots, increase mortality risk, heat and chemical related psychological stress, and opportunities for human‐wildlife interactions (Bateman et al. 2023; Birnie‐Gauvin et al. 2016; Coffin 2007). Similarly, buildings can function as ecological traps where anthropogenic subsidies (e.g., food, nest or roost site) increase area attractiveness despite the increased opportunity for negative human‐wildlife interactions and mortality (Hager et al. 2013; Vlaschenko et al. 2019). These risks associated with anthropogenic pressure and development can, subsequently, have trickle down effects on reproductive success (Corsini and Szulkin 2025). Thus, this produces a complicated decision‐making landscape in which maladaptive decisions have the potential to negatively impact fitness outcomes.

Despite the high‐risk, complex decision‐making landscape urban areas produce, many species have been able to persist, likely aided in part by adaptive reproductive decisions. Coyotes ( *Canis latrans* ) are one such species commonly found in cities, inhabiting nearly every large metropolitan area in the United States and Canada (Breck et al. 2019). As coyotes use dens to safeguard their offspring, the location and security of the den directly impact early life stage survival and ultimately population dynamics, suggesting that these decisions are critical to population persistence in highly dynamic urban environments. The demographic importance of coyote denning decisions has been demonstrated by previous research in rural areas, as anthropogenic mortality at dens (i.e., den hunting) was a common tool used to temporarily reduce local coyote populations (Connolly 1981; Robinson 1962; Till and Knowlton 1983; Young and Dobyns 1945). Like rural environments, it should be expected that den site selection can similarly drive population‐level trajectories, and yet little is known about these reproductive decisions in urban landscapes. To date, only two studies have directly assessed den selection in suburban (Way et al. 2001) or urban areas (Raymond and St. Clair 2023) while others have noted instances where dens have been observed (Dodge and Kashian 2013; Grubbs and Krausman 2009).

Because of the importance den sites are likely to play in individual fitness and population dynamics of urban coyotes, we aimed to determine how cover habitat, built infrastructure, and anthropogenic pressure impact coyote den site selection in the rapidly urbanizing landscape of Atlanta, GA. We predicted that coyotes would avoid both built infrastructure and anthropogenic pressure while selecting for cover as a means to mitigate risks associated with denning. Our secondary aim was to assess the structural components of urban coyote dens. We predicted coyotes would use various den structures including those that incorporate anthropogenically derived materials. Ultimately, our goal was to establish a baseline understanding of urban coyote denning in Atlanta and highlight the role these decisions may play in their population dynamics.

## 2 Study Area

**FIGURE 1:**
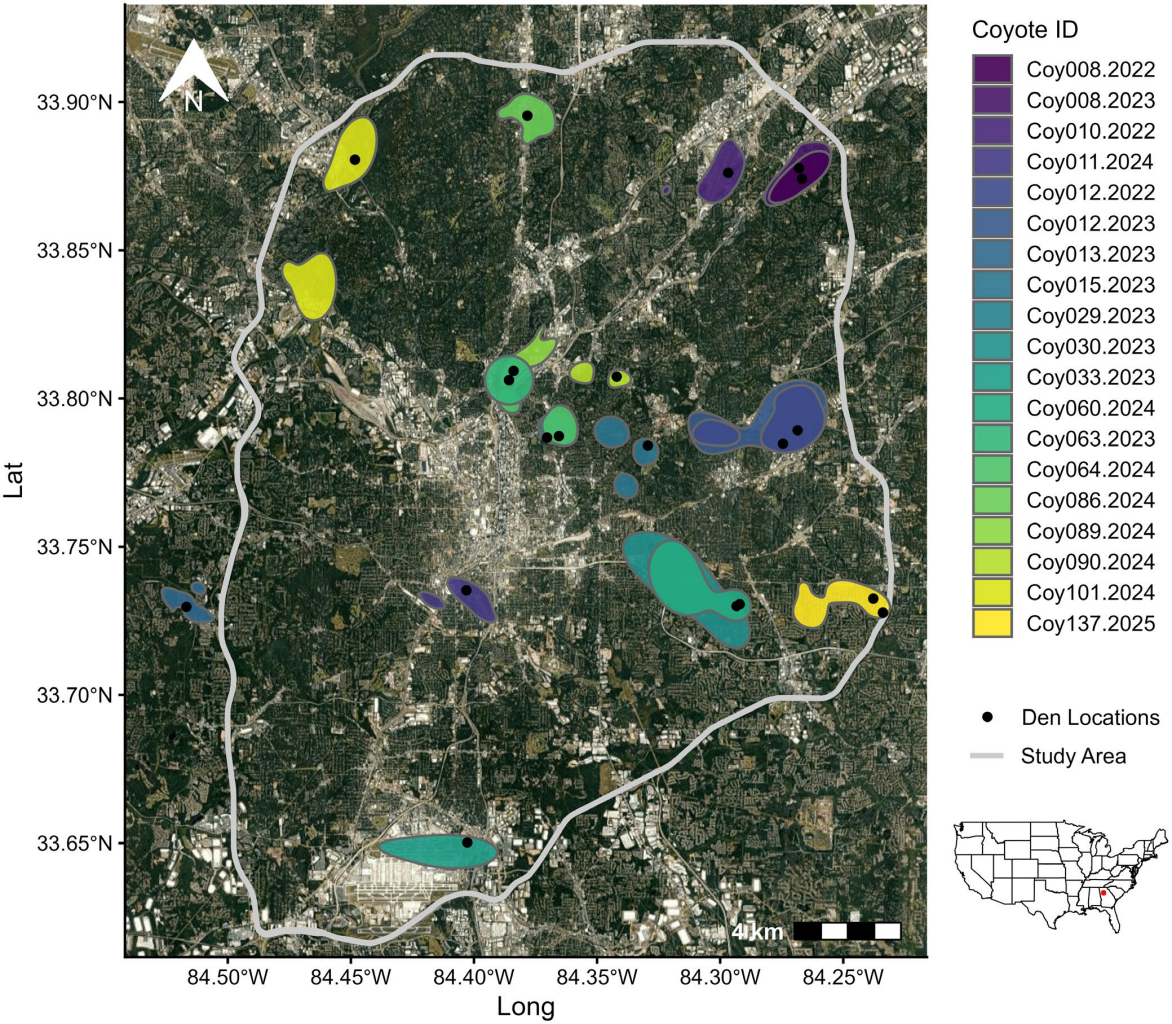
The study area was located in northwestern Georgia and was bound by Interstate 285 that surrounded Atlanta. Map depicts 50% AKDE (autocorrelated kernel density estimate) home ranges (i.e., core areas) for denning coyotes in Atlanta, GA from 2022 to 2025. All coyotes denned in core areas and average core area size was 3.7 km^2^ (range: 1.06–9.45km^2^). Coyote ID represents the parent ID and the year the den was located.

We conducted our research in metropolitan Atlanta, GA, home to approximately 6.2 million residents. We constrained our study area to within the boundaries of I‐285 where population density was ~2768 people/km^2^ (Figure 1). Impervious surface levels were highest downtown with infrastructure built along interstates that radiated from the center. Outside of this, the landscape was predominantly single‐family homes which made up 38% of total housing units. The greater Atlanta area had the highest percentage of tree canopy cover (47.9%) of any large US city (Giarrusso and Smith 2014). The Chattahoochee River National Recreation Area ran along the northwest boundary of the study area providing significant forest cover. The climate was categorized as hot and humid with an average yearly rainfall of 132 cm and seasonal temperatures from 1°C to 31°C. Elevation ranged from 225 to 320 m.

## 3 Methods

### 3.1 Capture and Den Identification

We captured adult coyotes from January to April in 2022–2024 using two coiled MB‐550 offset jaw foothold traps (Minnesota Trapline Products, Model: MB550RCO, Pennock, Minnesota, US). We continuously monitored each trap with a cellular wildlife camera (Spartan Camera, Model: GL‐VLTEb, Duluth, Georgia, US) and responded within 2 h of capture. Coyotes were restrained with a catchpole, leg hobbles, and a muzzle. Each coyote was weighed, sexed, aged, and fitted with a GPS collar (VECTRONIC Aerospace Vertex Lite 1‐D Iridium, Berlin, Germany or LOTEK InSight S, Newmarket, Ontario, Canada). We used tooth wear and reproductive condition to place coyotes into juvenile (0–1 year), subadult (1–2 years), or adult (2+ years) age‐classes (Gier 1968). Collars were programmed to record GPS locations every 5 h for 2 years. Fix rate increased to every 2.5 h during January, April, July, and October to meet the objectives of a concurrent study.

To locate dens, we used spring GPS clustering to identify potential den sites for field confirmation. We defined a GPS cluster as five or more repeated locations within 60 m that spanned across 7 days. These often formed a starburst pattern where most locations were concentrated around a central location (e.g., potential den) with short bouts away from the site, presumably for foraging. Upon visiting a GPS cluster, we used omni‐directional searching to identify potential dens starting from the central location. Each searching attempt included a minimum of three trained wildlife professionals. Indicators of coyote dens included recent digging, holes or tunneling in the earth, vegetation, or anthropogenic structures, hair on roof entrance, tracks, absence of insects, nearby scat, trailing into entrance, or smoothed dirt. Potential dens were searched for pups via flashlight or 7.6 m long burrow camera (Smith et al. 2005). All den checks were completed between April 4th and 29th. Though some possible unoccupied dens, which included at least one of the aforementioned den indicators, were located during our search, we were unable to rule out their use by other mesocarnivore species. Thus, we only included dens with visual confirmation of pups in the analyses as we did not want to introduce Type 1 error.

When possible, pups were removed from the den and processed. Each pup was weighed, sexed, and uniquely marked with a subcutaneous PIT tag between the shoulder blades along the dorsal midline (Avid Identification Systems, Model: AVID2028, Norco, California, US). We estimated pup age to the week through weight, tooth eruption, and ear erection. Coyote capture and handling was approved by the Institutional Animal Care and Use Committee at the University of Georgia (Protocol Numbers: A2021 07‐021‐Y3‐A4, A2024 07‐020‐Y1‐A0).

### 3.2 Den Structure Characteristics

To determine coyote use of various structures, we classified dens into dirt mound, hillside burrow, flat ground burrow, culvert, root ball, fallen hollow tree, standing tree trunk, concrete, and/or man‐made structure. We also recorded additional descriptions of the structure, such as tree species, material (e.g., concrete vs. metal culvert), and configuration (e.g., burrows with multiple chambers or entrances). Den depth and entrance orientation, height, and width were measured. Given data limitations and extreme structure variability, we grouped dens into either natural (e.g., ground burrow, tree trunk) or anthropogenic (e.g., concrete, over‐turned boat). Any dens that included both features (e.g., ground burrow under abandoned concrete slab) were assigned to their predominate structure type. For instance, though there was a ground burrow in the aforementioned example, that den would be classified as anthropogenic because 50% or more of the den structure was made from anthropogenic material.

### 3.3 Landscape Characteristics

We assessed the impacts of natural habitat, built infrastructure, and anthropogenic pressure on den site selection. Due to the limited sample size of active den sites, we only included predominant landscape features as covariates, specifically: distance to cover habitat, percent impervious surface, and distance to buildings, respectively. Building and cover habitat layers were extracted from a 30 × 30 m OpenSteetMap enhanced landcover classification raster (Gelmi‐Candusso et al. 2024). Cover habitat was a reclassified combination of the original landcover classes of “dense forested green area” and “heterogeneous green” (e.g., scrub and brush). For each landcover class, we calculated the Euclidean distance to the closest habitat class for each 30 × 30 m pixel on the landscape. We extracted impervious surface from the National Land Cover Database (U.S. Geological Survey 2024). This is calculated as the percentage of land covered by impervious surfaces within a given 30 × 30 m pixel. Because these calculations give insight into the proportions of permeable and impermeable area within a given pixel, they are often used as a relative measure for development. Each covariate was centered and scaled before analysis.

### 3.4 Den Site Selection Analyses

To assess den site selection, we used a 3rd order resource selection function design which operates in a used vs. available framework (Manly et al. 2007). We expected that coyotes would den inside their core areas, thus to delineate available locations, we first calculated 50% autocorrelated kernel density estimator (AKDE) home ranges (i.e., core areas) for each coyote in each den year using the ctmm package (Calabrese et al. 2016). Fifty percent isopleths represent the space an animal utilizes 50% of the time (Chan et al. 2022) and are commonly used in core area calculations (Abril‐Colón et al. 2022; Lee et al. 2022; Silva‐Opps and Opps 2011). Because AKDEs account for autocorrelation in GPS locations, we avoid arbitrary core area shrinkage that may mask core area importance. ADKEs also efficiently use variable fix rates for accurate home range estimation (Calabrese et al. 2016), thus we used both 2.5 and 5‐h locations in home range calculations. We only included locations outside of den preparation and pup rearing season (Mar–Aug. in Georgia; unpublished data) for a given den year. This reduced bias in our home range estimates because it encompassed the total extent of habitat available for den site selection, while excluding the high density of locations that occurred near the den site during den preparation and pup rearing. For one individual (coy063) that did not have GPS locations during the unbiased sampling period, we generated a circular polygon that was median core area size and centered it on the den location. When working in a used vs. available framework, it is important that the available locations represent the true available space (Benson 2013; Street et al. 2021). Thus, we systematically sampled every 30 m pixel within the core area for each coyote in each den year.

We assessed collinearity among covariates using both Pearson's pairwise correlation and variance inflation factors (VIF). No covariates had *r* > 0.7 and all VIF were approximately 1. We used the clogit function from the survival package to run a global conditional logistic regression model (Therneau 2024). This structure includes a stratification within the data to compare the used den site to only the available sites for that individual within that year. We then calculated the Relative Selection Strength (RSS) for each covariate to account for the fact that individual dens occur within different core areas comprised of differences in landscape covariates (Avgar et al. 2017). All analysis was conducted in program R (R Core Team 2021).

## 4 Results

We GPS‐collared 48 coyotes (25 F, 23 M), of which 32 were adults, 11 subadults, and 5 juveniles. From the collared individuals, we located 20 dens where pups were present, resulting in 99 pups being processed. Based on age of pups during den checks and timing of spring clustering, we determined that Georgia coyotes give birth from mid‐March to mid‐April. Litter size ranged from 2 to 9 ($\bar{x}$ = 5.5 ± 1.95) pups per litter with nearly a 1:1 sex ratio (49 M, 50 F). Weight ranged from 0.2 kg to 2.3 kg depending on the age of pups when the den was located.

### 4.1 Den Structure

**FIGURE 2:**
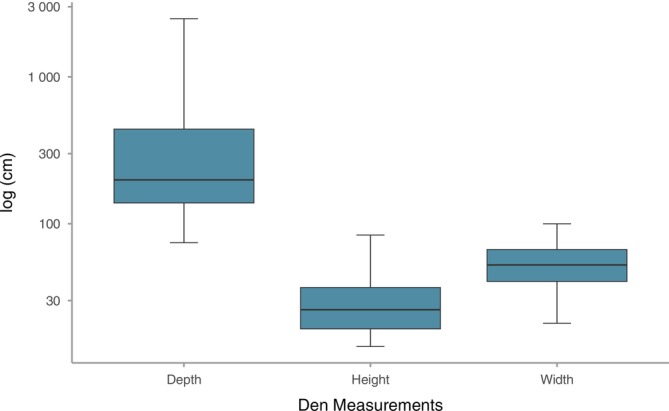
Size of coyote dens in Atlanta, GA measured from 2022 to 2025. Depth was measured from den entrance to back and height and width were measured at den entrance. Units (i.e., centimeters) are log scaled to highlight variation in size with den depth having the greatest variance.

**FIGURE 3:**
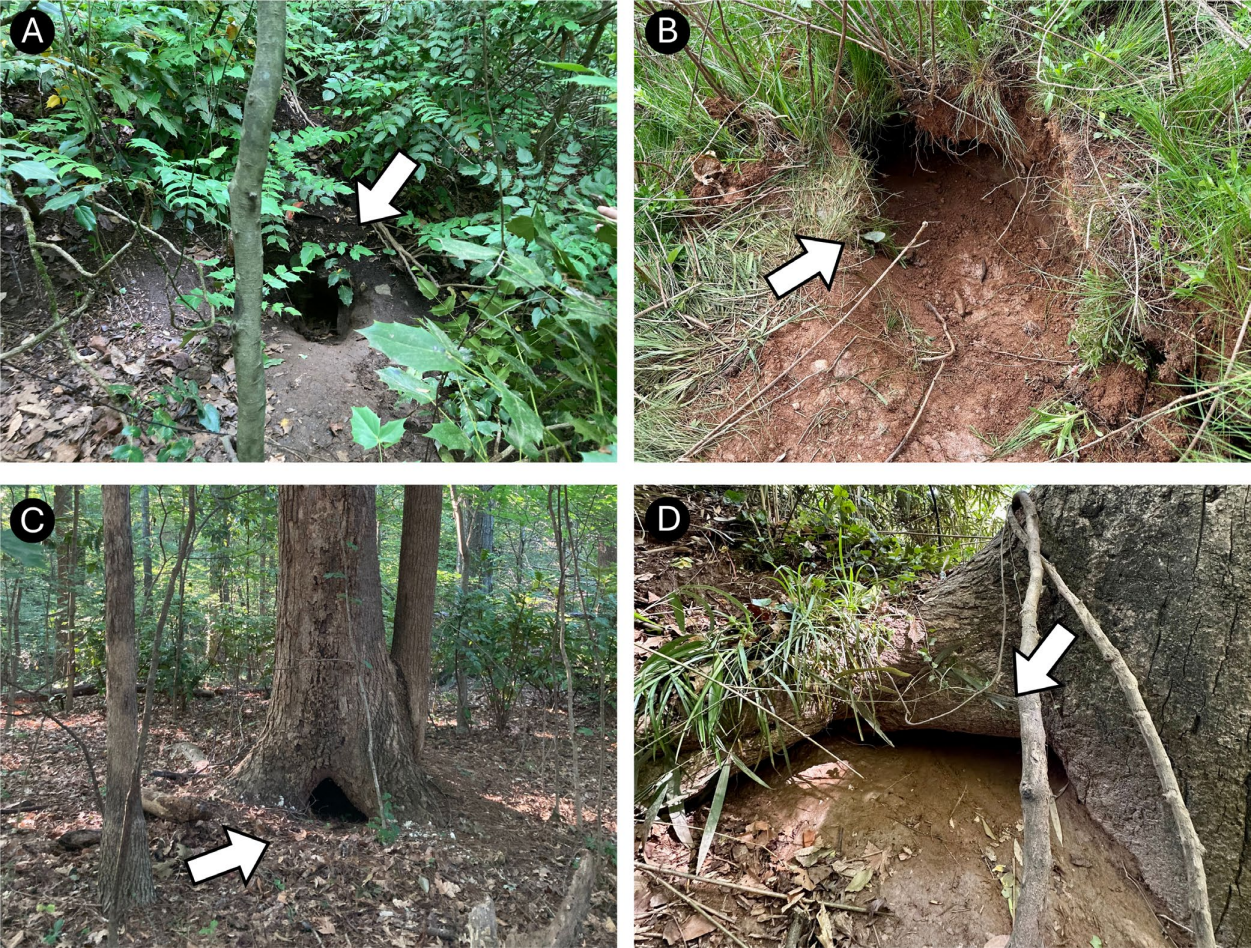
Examples of dens in Atlanta, Georgia classified as natural structure. White arrows signify den entrance. (A, B) Ground burrow. (C) Standing tree trunk. (D) Hillside burrow where root of standing tree was used as roof of den for full length of burrow.

**FIGURE 4:**
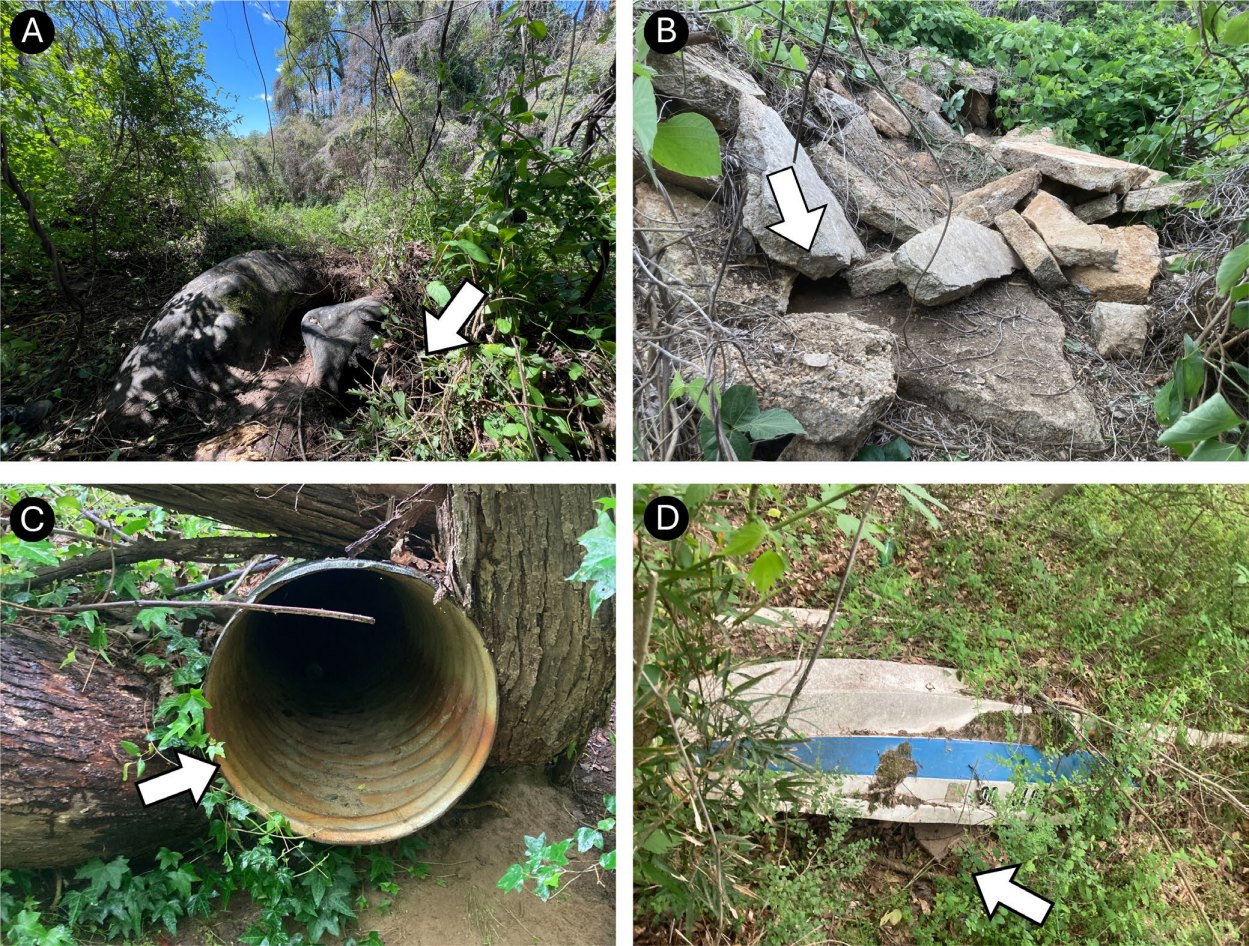
Examples of dens in Atlanta, Georgia classified as anthropogenic structures. White arrows signify den entrance. (A) Large tractor tire half buried under ground with opening dug out. Offspring were in the tire wall. (B) Pile of discarded concrete with multiple entrances with one main burrow where offspring were found. (C) Abandoned metal road culvert. (D) Abandoned overturned boat. Offspring were tucked in the stern of a boat that was semi‐buried.

Den size varied by measurement type (Figure 2). Mean den depth was 454.2 cm (±161.5 cm), with individual depth being a function of the den structure. For instance, the deepest den structure was an abandoned road culvert (24.9 m) whereas a standing tree trunk depth can only be as deep as the tree's width. Average den width (55.05 cm ± 5.7) and height (31.0 cm ± 4.6) had lower variation overall (Figure 2). Fifty‐five percent (*n* = 11) of dens were in natural structures, which included flat ground and hillside burrows as well as standing and fallen tree trunks (Table S1 and Figure 3). Anthropogenic dens (45% of dens) included discarded concrete, an overturned boat, and a tractor tire (Table S1 and Figure 4). Two‐thirds (*n* = 6) of the anthropogenic dens included at least some concrete features.

### 4.2 Den Site Selection

**FIGURE 5:**
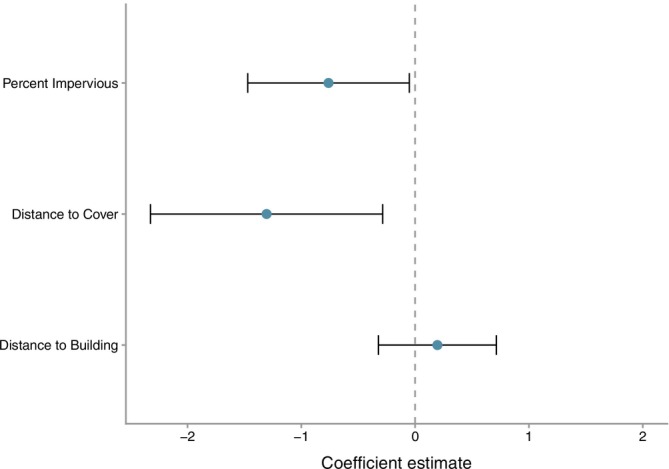
Coefficient estimates and associated 95% confidence intervals for each covariate in the coyote den selection model developed for Atlanta, GA. Confidence intervals overlapping zero are not significant. Covariates to the left of zero have a negative relationship whereas to the right of zero have a positive relationship.

All coyotes denned inside their core area (Figure 1). Average core area size was 3.7 km^2^ (range: 1.06–9.45 km^2^). Inside these core areas, coyotes strongly selected for den sites that were near cover (distance to cover habitat: β = −1.30 ± 0.52, *p* = 0.012; Figure 5). Thirty‐five percent of dens (*n* = 7) were located inside cover habitat and 50% of all dens (*n* = 10) were within 10 m of cover habitat. Only 1 den was greater than 1 km away from cover habitat. In addition, coyotes avoided den sites with high levels of impervious surfaces (β = −0.76 ± 0.36, *p* = 0.036, Figure 5). Average distance from a den to a building was 135 m (range: 12.93–505.56 m). However, distance to building was not significant (β = 0.20 ± 0.26, *p* = 0.459, Figure 5).

## 5 Discussion

Urban coyotes must make fitness optimizing decisions in a highly dynamic and risky landscape and such decisions regarding denning are critical for early life stage survival. In Atlanta, we found that coyotes were selecting areas that likely conferred increased fitness outcomes relative to anthropogenic pressure and resource availability. At the landscape scale, all sampled coyotes selected den sites within their core areas. At the 3rd order scale, coyotes selected cover habitat and avoided areas of high percent impervious surfaces (e.g., roads, parking lots, etc.). Additionally, coyotes demonstrated extreme flexibility in den structure use (i.e., natural or anthropogenic) and tended to incorporate hard organic (e.g., large tree roots) or inorganic (e.g., concrete) material into the structure, presumably to increase structural soundness and protection. Cumulatively, these decisions highlight the ability of coyotes to navigate complex decision‐making landscapes to potentially benefit reproductive success.

In Atlanta, we observed coyotes making reproductive decisions specifically related to risk mitigation that are likely to increase reproductive success. First, every coyote denned inside their core area, demonstrating a collective population‐level decision. This is not surprising as core areas represent the most utilized space within an animal's home range (Samuel et al. 1985) and subsequently have the strongest cognitive mapping which increases risk predictability, ultimately improving animal survival (Spencer 2012). Core areas also tend to have a closer proximity of resources and further distance from risk (e.g., intraspecific interactions at territory edges), both of which contribute to improved reproductive success (Smith et al. 2015). Within their core area, coyotes in Atlanta generally avoided human‐altered spaces, as measured by impervious surfaces, when selecting den sites. This follows similar results from Edmonton, Canada where coyotes avoided areas of high impervious surface for denning (Raymond and St. Clair 2023). Other studies of urban coyotes have similarly demonstrated a general spatial avoidance of impervious surfaces (Gese et al. 2012; Hursh et al. 2023). Thus, it is not surprising that, during a critical reproductive period, coyotes would strongly avoid these locations for denning, given that they present limitations for den building and include high risk areas, such as roads, parking lots, and large commercial complexes.

**FIGURE 6:**
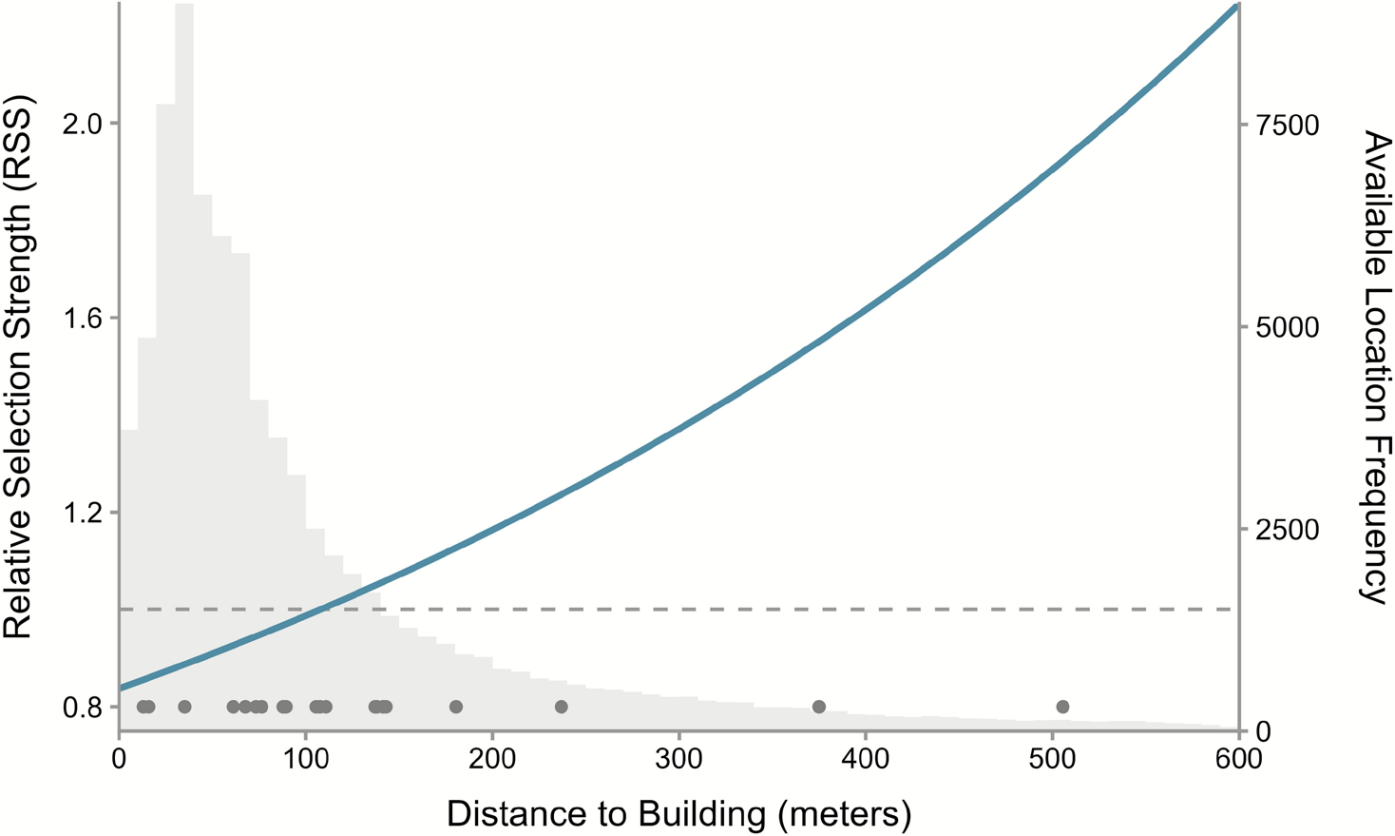
Relative selection strength of distance to buildings for coyote dens in Atlanta, GA. Values below 1 signal avoidance and values above 1 signal selection. The relationship is not statistically significant. Gray histogram represents frequency of available locations and their associated distance to building values. Available locations were sampled from every 30 m pixel inside coyote core areas. Used den sites are represented by dots along the x‐axis.

Although coyotes avoided human‐altered landscapes, we did not observe avoidance of or selection for buildings themselves. Given that coyotes tend to avoid areas of high impervious surface, our results related to distance to buildings may simply be an artifact of context‐specific risk levels. For example, the building type (e.g., industrial vs. residential), occupancy status (e.g., occupied, unoccupied, abandoned), and surrounding cover habitat likely concurrently impact the perceived riskiness of individual buildings, and thus the probability of denning close to a specific building. These results indicate that coyotes are likely assessing risk subtleties at much finer spatio‐temporal scales than we can quantify using currently available spatial information (i.e., geographic information system spatial layers). This is an important distinction because many parts of the urban landscape are in close proximity to buildings. In fact, inside the core areas of sampled coyotes, most locations were within 150 m of a building (Figure 6). While this likely prevents coyotes from physically avoiding buildings at the scales that we measured, it may also benefit urban coyotes by providing constant knowledge of the dynamic risk associated with individual buildings. Together, this underscores an important nuance in animal decision‐making that is likely complicating our ability to detect wildlife responses within urban landscapes.

Beyond avoiding high risk areas, we also observed coyotes making reproductive decisions that may contribute to increased offspring survival and reproductive success. First, we observed that coyotes strongly selected den sites close to or inside cover habitat (i.e., forested and shrub; Figure 5). More specifically, 50% of all dens were within 10 m of cover habitat emphasizing the importance of cover to conceal dens from conspecifics, humans, and their pets. The use of concealment (vegetation, configuration, etc.) for dens and den entrances is common for coyotes in rural areas (Althoff 1979; Hallett et al. 1985; Kamler et al. 2005), which suggests that this decision may be a constraining habitat condition for coyotes in urban areas as well. Additionally, coyotes demonstrated behavioral plasticity when selecting den structures which allowed them to take advantage of both natural (55%) and anthropogenic structures (45%). Importantly, all the anthropogenic structures in our study were abandoned or discarded suggesting that coyotes were choosing the most protective structure available to them regardless of whether it was composed of man‐made material or not, particularly if it also provided high concealment. In doing so, coyotes are making fine‐scale decisions on both location and structures to protect their young and increase the probability of reproductive success.

Our results mirror den site selection behaviors identified in Edmonton, Canada in that both studies found no significant effect of distance to buildings, significant avoidance of impervious surfaces for denning, and frequent use of anthropogenic material in den structures (Raymond and St. Clair 2023). Surprisingly, Raymond and St. Clair (2023) interpreted these results to suggest that coyotes are tolerant of anthropogenic presence. In contrast, we would argue that although coyotes demonstrated high use of anthropogenically produced material in den structures, they are likely attempting to avoid anthropogenic risk at dens through simultaneous selection for cover habitat and avoidance of areas with a high percent of impervious surfaces (e.g., roads, development). In fact, the use of these structures should be considered a technique to protect offspring as concrete and other anthropogenic materials can be at least as strong and stable as naturally occurring structures. Simple integration of anthropogenic material into den structures does not signal tolerance of anthropogenic presence or pressure but highlights coyote behavioral plasticity in risk mitigation.

While urban coyotes are making reproductive decisions to mitigate risk, human‐coyote interactions may counteract these benefits. Conflicts resulting from negative human‐coyote interactions can have cascading impacts on coyote fitness as retaliation to human‐carnivore conflicts often results in the removal of parents and ultimately offspring (Lorand et al. 2022). Risks associated with conflicts (e.g., increased stress, mortality) are present throughout the year; however, coyotes are faced with greater fitness ramifications during denning. Raymond and St. Clair (2023) observed this paradigm where human‐coyote conflicts were more likely to occur in areas closer to den sites during the denning period. This is likely due to the combination of an evolutionary fitness optimization technique employed to protect offspring from conspecifics and apex predators (i.e., den defense) as well as the reduction of movement and congregation of group members around den sites for pup rearing. However, though evolutionary advantageous, such a technique may be becoming maladaptive in urban areas, as retaliation to a conflict may result in removal. Nevertheless, coyotes today must balance intrinsic behaviors and environmental and physiological constraints with anthropogenic risk which our results suggest they do through reproductive decision‐making.

In this study, we begin to fill a critical gap in the knowledge of urban coyote denning decisions by assessing the impact of landscape characteristics on den location, establishing baseline knowledge of structure use, and drawing attention to the risk nuances that urban coyotes must navigate when making denning decisions. While our study highlights the role these decisions could potentially play in generating positive fitness outcomes and regulating population dynamics, we were unable to determine how those decisions impacted overall fitness for coyotes in Atlanta. This is because we assessed only one component of this decision‐making process (i.e., den site selection), whereas many factors may cumulatively determine coyote reproductive success, such as disease burden, maternal age, and parental experience. Thus, to better understand the linkage between reproductive denning decisions and population demographics, future research should aim to directly assess the impacts of structure type and den location on pup survival and reproductive success.

As urban areas grow and green cover continues to be converted to impervious surfaces, our results indicate that urban coyotes will face increasingly challenging denning decisions. As cover areas become smaller and less abundant, coyotes will be forced to make maladaptive denning decisions which put them at risk for decreased reproductive success and increased human‐wildlife conflict. This will have implications for population dynamics and will likely shape evolutionary processes for urban coyotes. More broadly, unchecked land conversion could have significant evolutionary impacts for site‐obligate species that are less adaptive or highly specialized. Thus, we call for more monitoring of reproductive decision making in urban areas to expand our understanding of how our land use changes are impacting population dynamics and evolutionary processes across taxa.

## Author Contributions

**Summer Fink:** conceptualization (lead), data curation (lead), formal analysis (lead), funding acquisition (equal), investigation (lead), methodology (lead), project administration (lead), visualization (lead), writing – original draft (lead), writing – review and editing (lead). **Daniela Guerrero:** data curation (equal), writing – review and editing (equal). **Eden Nitza:** data curation (equal), writing – review and editing (equal). **Michel Kohl:** conceptualization (supporting), funding acquisition (lead), project administration (supporting), resources (lead), supervision (lead), writing – review and editing (supporting).

## Conflicts of Interest

The authors declare no conflicts of interest.

## Supporting information


**Table S1:** Coyote den structures, descriptions, and measurements from sampling completed 2022–2025 in Atlanta, GA.

## Data Availability

The R scripts and data required to reproduce analyses are available in the Zenodo repository https://doi.org/10.5281/zenodo.17399512. Due to the sensitivity of coyote dens and denning spaces, those raw GPS coordinates are only available on request from the corresponding author.
