# Supplementary figures and images for: Coyotes Choose Cover Over Concrete When Selecting Den Sites
Source: Ecol Evol. 2026 Feb 27;16(3):e73186. doi: 10.1002/ece3.73186 (PMC12949333; doi:10.1002/ece3.73186)

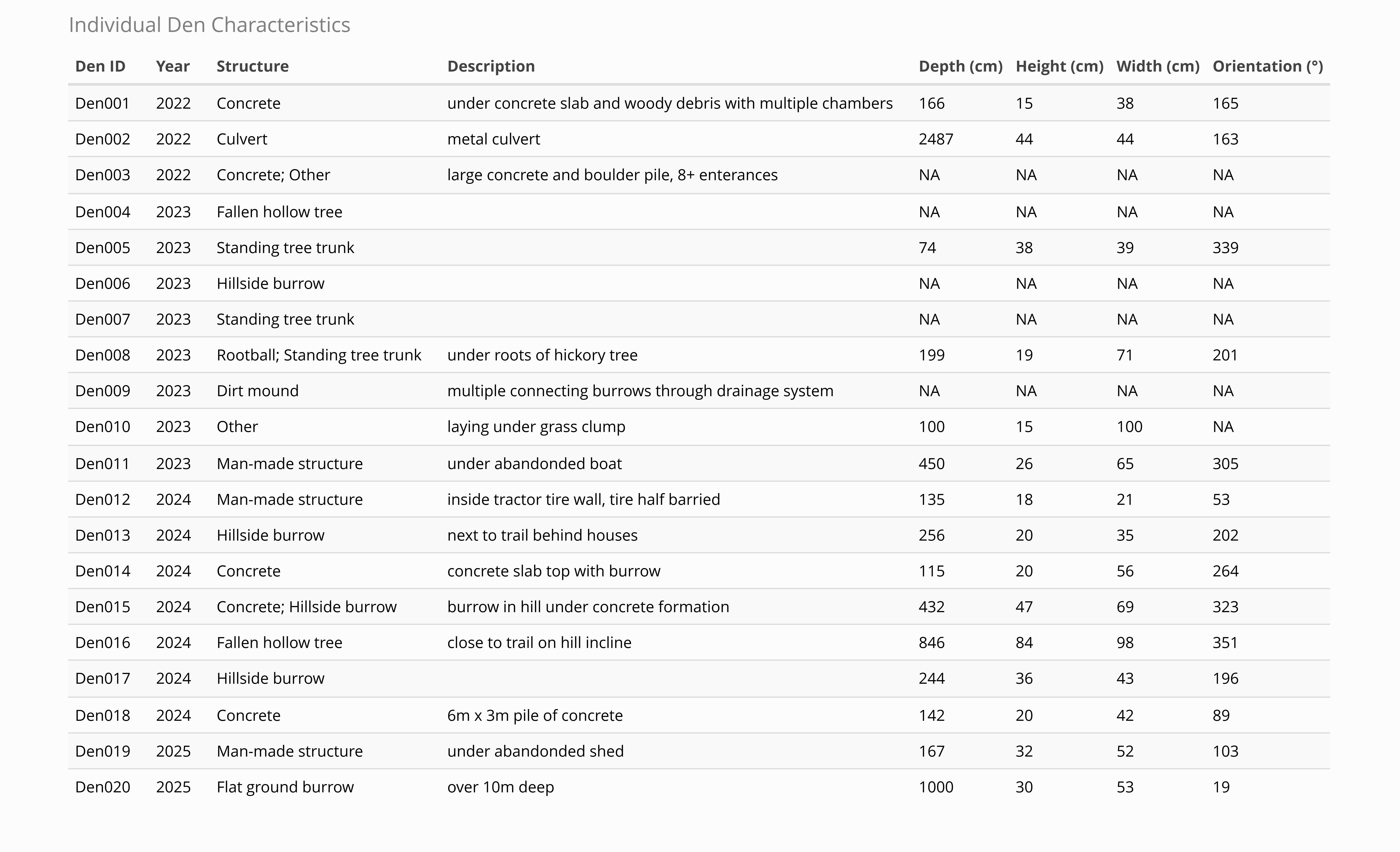

Supplement: Supplementary file 1 — Table S1: Coyote den structures, descriptions, and measurements from sampling completed 2022–2025 in Atlanta, GA. [file ECE3-16-e73186-s001.png]
